# Supplementary material for: Granatum: a graphical single-cell RNA-Seq analysis pipeline for genomics scientists
Source: Genome Med. 2017 Dec 5;9:108. doi: 10.1186/s13073-017-0492-3 (PMC5716224; doi:10.1186/s13073-017-0492-3)
Supplement: Additional file 1: — Supplementary Figures S1, S2, and S3. (PDF 307 kb) [file 13073_2017_492_MOESM1_ESM.pdf]

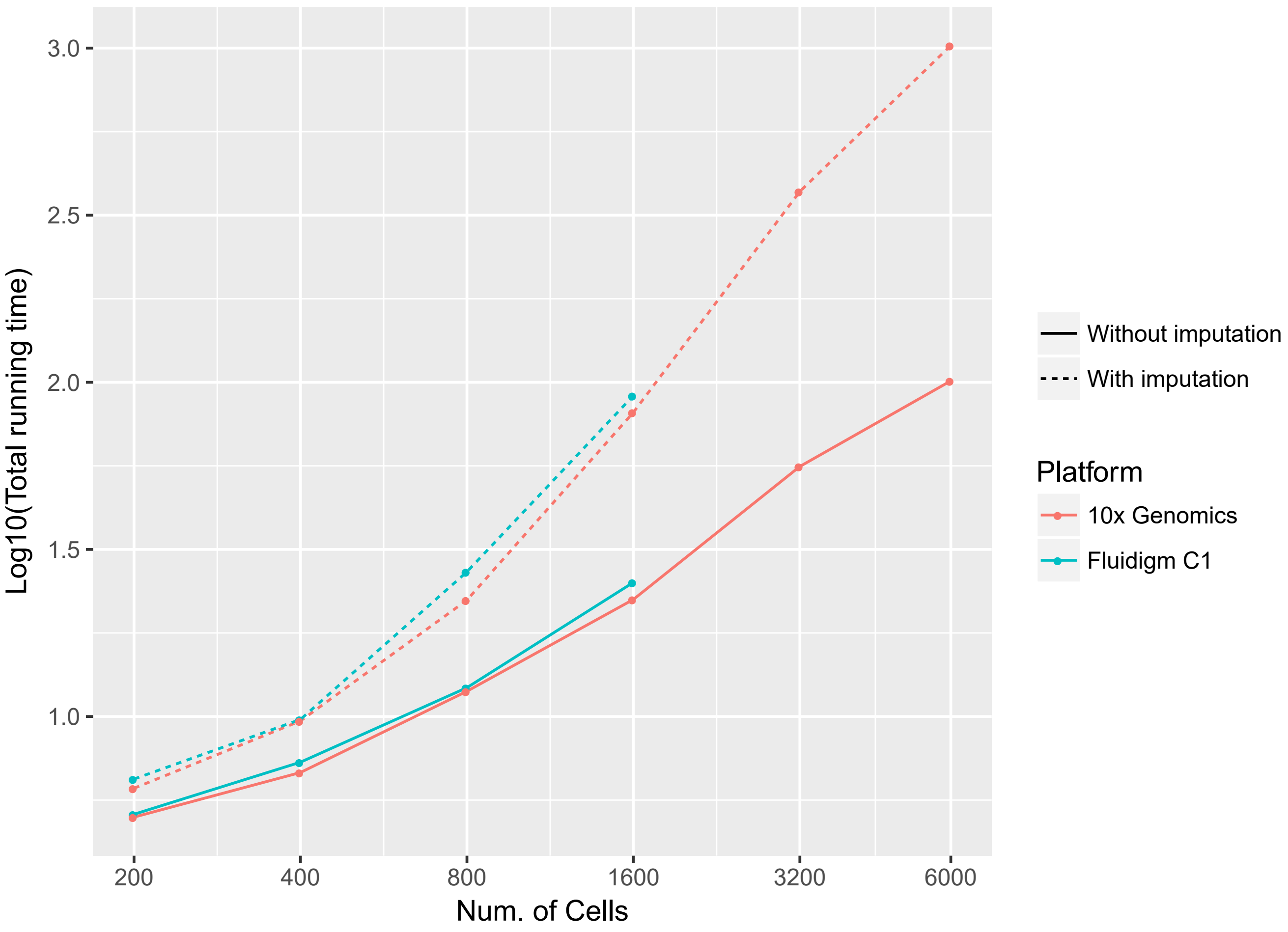

Suppl. Figure 1: Granatum total running time(in log10 scale)with various numbersof cells.

Datasets with various sizes from two single-cell platforms (Fluidigm C1 and 10x Genomics) are used. To generate expression data up to6000cells, the Fluidigm C1 datasets are simulated using Splatter, with parameters estimated from the K-dataset(118cells). The 10x Genomics datasets are down-sampled from the original6000-cell PBMC dataset. The x-axis represents the size of the dataset, and the y-axis represents the total running time (in minutes) of Granatum.The imputation step (scImpute) takes about90% of the total running time, and Monocle based pseudo-time construction step takes about 80% ofthe rest of the running time.

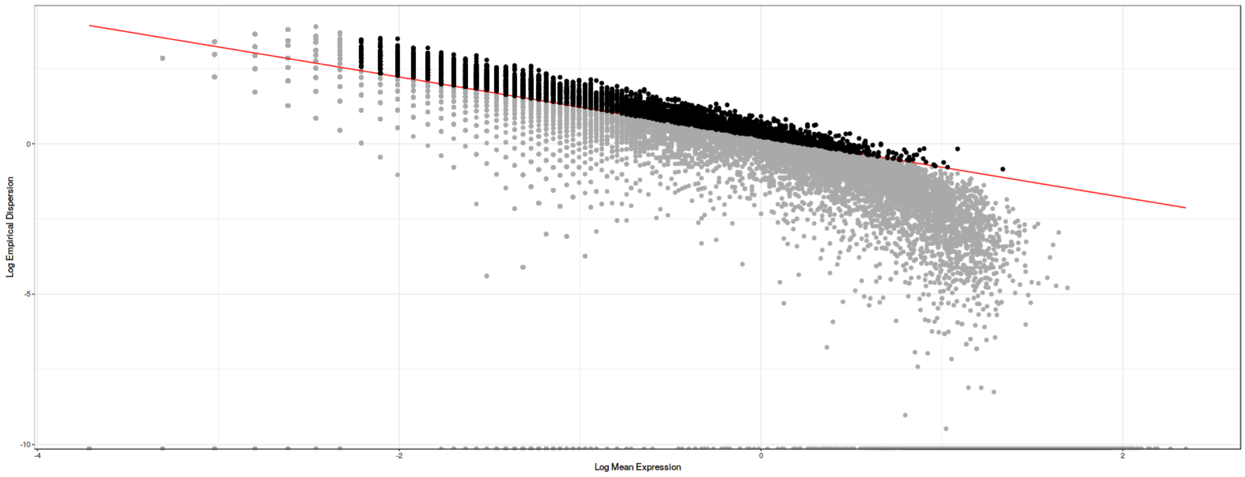

Suppl. Figure 2: The Gene filtering step. The y-axis of the scatter-plot is the empirical dispersion, estimated by a negative binomial model. The x-axis is the log mean expression of each gene.The red line is the fit of a negative binomial model onto the data.Black points represent gene to be kept and gray points are filtered genes.

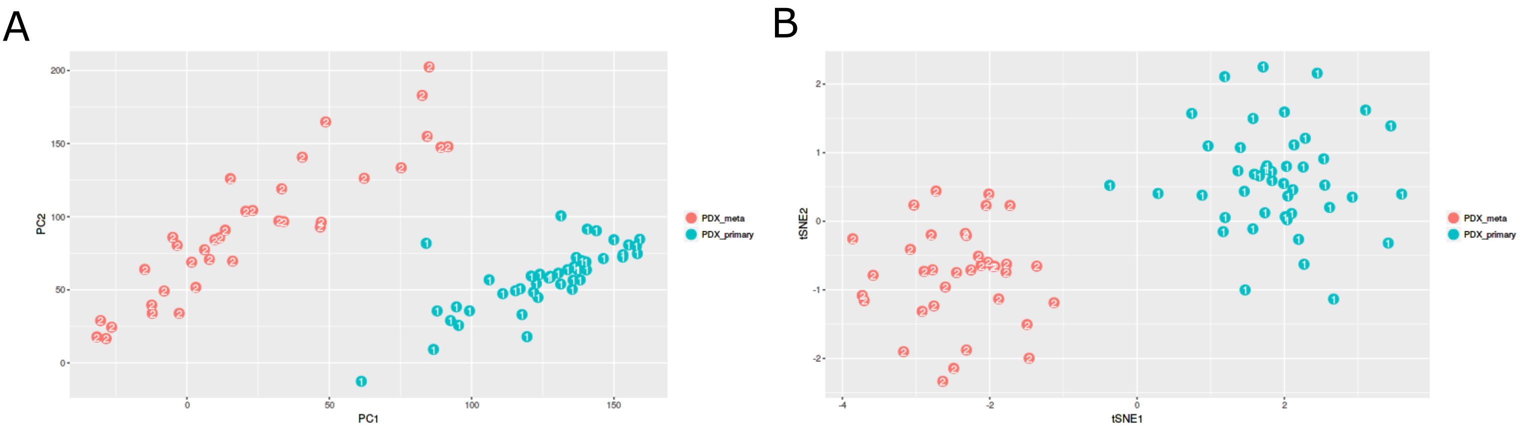

Suppl. Figure 3: The Clustering step.(A) PCAand (B) Correlation t-SNE plots of single cells (dots) are shown,with colors indicatingthe cell types reported in the original dataset and cluster number (1,2) super-imposed on the cells.
